# Prevalence and Determinants of a Blunted Parathyroid Hormone Response in Young Saudi Women with Vitamin D Deficiency: A Cross-Sectional Study

**DOI:** 10.1155/2021/5579484

**Published:** 2021-09-17

**Authors:** Hanan Al Kadi

**Affiliations:** Department of Physiology, Faculty of Medicine, King Abdulaziz University, Jeddah, Saudi Arabia

## Abstract

Vitamin D deficiency is highly prevalent among the Saudi population. Increased parathyroid hormone (PTH) secretion is an appropriate homeostatic response to correct the resultant hypocalcemia. However, not all vitamin D deficiency patients have increased PTH levels. This study determined the prevalence of a blunted PTH response to vitamin D deficiency among apparently healthy young Saudi women and assessed anthropometric and biochemical factors associated with this response by performing a secondary analysis of data obtained from a cross-sectional study conducted at the “Center of Excellence for Osteoporosis research.” Overall, 315 women (aged 20–45 years) with vitamin D deficiency (serum 25-hydroxyvitamin D (25(OH)D) levels <30 nmol/L) were included. They were divided into two groups according to the laboratory cutoff value of PTH (<7 or ≥7 pmol/L), and anthropometric and biochemical characteristics of both groups were compared. Women with a blunted PTH response (*n* = 62, 19.7%) had a significantly lower body mass index (BMI) (*P* < 0.001) and smaller waist circumference (*P*=0.001). They also had significantly higher serum 25(OH)D (*P*=0.001), corrected serum calcium (*P* < 0.001), and phosphate (*P*=0.003) levels than those with an elevated PTH response (*n* = 253, 80.3%). Multiple logistic regression analysis showed that lower BMI (OR = 0.925; 95% CI: 0.949–0.987) and higher 25(OH)D (OR = 1.068; 95% CI: 1.014–1.124) and serum calcium (OR = 8.600; 95% CI: 1.614–45.809) levels were significantly associated with a blunted PTH response (*R*^2^ = 0.178). A blunted PTH response to vitamin D deficiency is mainly observed among women with lower BMI. Higher serum calcium and 25(OH)D levels and lower BMI were significant predictors of a blunted PTH response, which may indicate that these subjects are adapting to lower 25(OH)D levels and maintaining normal calcium levels without the need to increase PTH secretion. The mechanisms underlying this adaptation are unclear, and future studies to explore these mechanisms are warranted.

## 1. Introduction

Vitamin D deficiency is a widespread health problem among women living in Jeddah, Saudi Arabia. [1, 2]. The active form of vitamin D (1,25-dihydroxycholecalciferol) stimulates calcium absorption from the gut; thus, vitamin D deficiency leads to malabsorption of dietary calcium, leading to hypocalcemia. The appropriate homeostatic response to hypocalcemia is increased parathyroid hormone (PTH) secretion from the parathyroid glands. However, not all women exhibit this increase in PTH levels. This blunted PTH response has been termed “functional hypoparathyroidism” [3]. This blunted response has been explained by different mechanisms, including low plasma magnesium level [4, 5], high dietary calcium intake [6], local adaptive mechanisms regulating intestinal calcium absorption (independently of vitamin D and PTH secretion), the so-called “intestinal calcistat” [7], and diurnal variations in PTH secretion [8]. Postmenopausal women with functional hypoparathyroidism exhibit higher bone mineral density than women with an elevated PTH response and, hence, may have lower fracture risks [9]. This is expected as PTH exerts an osteoclastic function on the bone [10] to correct hypocalcemia secondary to vitamin D deficiency. To the best of our knowledge, there are no data on the pattern of the PTH response among young women with vitamin D deficiency living in Jeddah, Saudi Arabia. Therefore, this study determined the prevalence of a blunted PTH response among otherwise healthy young Saudi women with vitamin D deficiency and assessed its associated anthropometric and biochemical factors.

## 2. Materials and Methods

**Figure 1:**
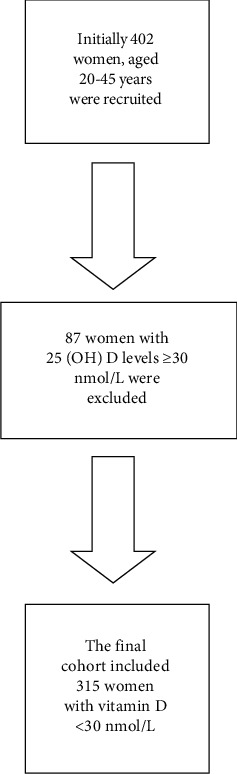
A flowchart of included and excluded women.

The current analyses were performed with data of women who participated in a cross-sectional study for the assessment of bone health at the “Center of Excellence for Osteoporosis research” (CEOR), Jeddah, Saudi Arabia. Details of the study design, recruitment, and data collection protocol have been described previously [11]. In brief, women were randomly recruited from 40 primary health care centers in Jeddah. Those willing to participate in the study were invited to visit the CEOR, complete a validated questionnaire, and undertake all the anthropometric and biochemical measurements. A subgroup comprising 315 women (aged 20–45 years) with serum 25(OH)D levels <30 nmol/L (vitamin D deficiency) [12, 13] was selected for this analysis (Figure 1).

All women who were included had no chronic diseases and were not on any long-term medications. Pregnant women were excluded. The weight, height, and waist circumference (WC) were measured in kg, cm, and cm, respectively. The body mass index (BMI, kg/m^2^) was calculated by dividing the weight by the square of the height in meters. Fasting blood samples were collected to determine 25(OH)D and PTH levels (in nmol/L and pmol/L, respectively). Both were determined by direct competitive chemiluminescence immunoassays using the LIAISON autoanalyzer (DiaSorin Inc, Stillwater, MN, USA). Other biochemical parameters (serum creatinine, total calcium, inorganic phosphate, magnesium, and albumin levels) were determined using the Vitros250 Chemistry Auto analyzer (Ortho Clinical Diagnostics, Johnson & Johnson Co., USA). The corrected total calcium concentration was calculated using the formula put forth by Payne et al.: “adjusted calcium concentration (mmol/L) = total calcium concentration (mmol/L) + 0.02 (40−serum albumin concentration (g/L))” [14]. Written informed consent was obtained from all participants, and the study was conducted as per the Declaration of Helsinki. This study was approved by the ethics committee of the CEOR.

### 2.1. Statistical Analysis

The distribution of the variables was assessed for normality using the Shapiro–Wilk test. All variables were not normally distributed except serum albumin. Continuous data are presented as medians (interquartile ranges) or means ± standard deviations according to the variable distribution. Categorical variables are presented as frequencies and percentages. Using the laboratory cutoff value for PTH, women were split into two groups: group 1—with a PTH concentration <7 pmol/L (blunted PTH response)—and group 2—with a PTH concentration ≥7 pmol/L (elevated PTH response). The two groups were compared with respect to different anthropometric and biochemical variables using the Mann–Whitney or Student's *t*-test depending on variable distribution. Spearman's correlation was used to determine the correlation between different variables. The chi-square test was performed to determine univariate associations between categorical variables. Multiple logistic regression analysis (via the enter method) was performed to examine the determinants of the blunted PTH response. Nagelkerke's *R*^2^ was reported to provide information about the explanatory ability of the model; odds ratios (OR) and their 95% confidence intervals (CI) were also provided. Statistical analyses were performed using SPSS version 20 (IBM Corp., Armonk, N.Y., USA). A *P* value <0.05 was considered statistically significant.

## 3. Results

This analysis included 315 women with vitamin D deficiency. A blunted PTH response (functional hypoparathyroidism) was prevalent among 19.7% (*n* = 62) of the women studied, while 80.3% (*n* = 253) exhibited an elevated PTH response. Thirty-five percent of the participants (*n* = 109) had severe vitamin D deficiency (25(OH)D level <12.5 nmol/L). The characteristics of the study participants are presented in Table 1. The differences in anthropometric and biochemical characteristics between women with a blunted PTH response and those with an elevated PTH response are also presented in Table 1.

**Figure 2:**
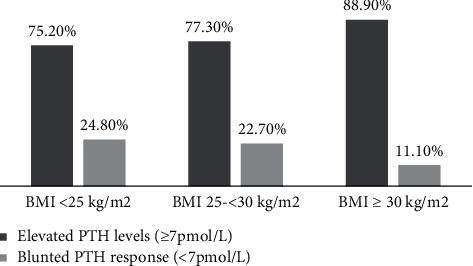
PTH responses among women with vitamin D deficiency according to BMI category (Χ^2^ = 7.512, *P*=0.023).

Women in the blunted PTH response group were significantly younger (*P*=0.049); had significantly lower BMI (*P*=0.001) and WC (*P*=0.001); and had higher 25(OH)D (*P*=0.001), calcium (*P* < 0.001), and phosphate (*P*=0.003) levels than those with an elevated PTH response. There were no significant differences concerning serum magnesium or creatinine levels.  Figure 2 shows the two groups according to the BMI category. The majority of women with obesity (88.9%) were categorized in the group with an elevated PTH response (Χ^2^ = 7.512, *P*=0.023).

Table 2 shows the correlation coefficients between the different variables among all women with 25(OH)D deficiency (*n* = 315). There was a statistically significant inverse correlation between PTH and 25(OH)D, corrected total serum calcium, and inorganic phosphate levels (*P* < 0.001 for all correlations), and a positive correlation between age (*P*=0.003) and measures of adiposity (BMI and WC) (*P* < 0.001 for both correlations).

Table 3 shows the results of the multivariable logistic regression analysis. The most significant variables, which were independently associated with a blunted PTH response, were higher corrected total serum calcium levels (OR = 8.600; 95% CI: 1.614–45.809), higher 25(OH)D levels (OR = 1.068; 95% CI: 1.014–1.124), and lower BMI (OR = 0.925; 95% CI: 0.949–0.987), as indicated by the odds ratio, with an *R*^2^ = 0.178.

## 4. Discussion

This study confirmed the previously described condition of “functional hypoparathyroidism,” in which a blunted PTH response is observed in some subjects, despite low 25(OH)D levels [9, 15–17]. The prevalence rate of a blunted PTH response was almost 20%, which was lower than that reported in other studies [16, 18–20], most of which defined vitamin D deficiency as a 25(OH)D level <50 nmol/L which is higher than the cutoff value used in this study (<30 nmol/L), and hence, the probability of a normal PTH level was higher. Moreover, several of these studies included older participants and postmenopausal women of different ethnic origins. PTH levels are known to increase with age [21], and ethnic differences in PTH secretion have been previously documented [22]. Therefore, it would be difficult to directly compare different studies of patients of different ethnic origins.

Higher serum calcium and 25(OH) D levels were the main determinants among participants with a blunted PTH response. These results may imply that these women could maintain calcium homeostasis despite the low 25(OH)D levels and without stimulating the parathyroid glands. One possible mechanism that was postulated by Garg is the so-called “intestinal calcistat” [7], which suggests a local mechanism whereby the body first adapts to increased “fractional calcium absorption” in the presence of low vitamin D status, independent of PTH secretion. If this local adaptation fails, secondary hyperparathyroidism ensues.

A low BMI was another predictor of a blunted PTH response. This agrees with the results of most studies that reported a direct relationship between increased adiposity and higher PTH levels [9, 15]. In this study, BMI was positively correlated with PTH but not 25(OH)D levels. Vitamin D deficiency is more prevalent among subjects with obesity [23] due to the sequestration of the fat-soluble vitamin D in fat stores, which reduces the bioavailability of 25(OH)D [24]. However, a previous study suggested that there exists a relationship between obesity and high PTH levels independent of the vitamin D status [25]. This is supported by the observation that PTH increases intracellular calcium levels [26], resulting in the inhibition of lipolysis in fat cells [27]. Therefore, lower PTH levels are expected to be associated with lower BMI in subjects without obesity. In fact, almost 90% of the subjects with obesity in this study belonged to the elevated PTH response group (Figure 2).

Magnesium deficiency is a well-documented cause of a blunted PTH response as it inhibits PTH synthesis and secretion [4, 5]. In this study, there was no statistically significant difference in serum magnesium levels between the two groups. However, serum magnesium level is not the best estimate of the tissue concentration of magnesium. The magnesium loading test would be the optimum measure, but it was not performed in this study.

Lower PTH levels were observed among female smokers [15], but this was not the case in the present analysis, probably owing to the low number of women who smoked (5.7%). In this study, smoking status was not associated with the PTH response in the multivariate analysis.

Kidney function is known to affect PTH levels [17]; however, serum creatinine was not independently associated with the PTH response in this study. This is probably because the cohort studied was healthy women with normal serum creatinine levels.

A major limitation of this study includes its cross-sectional design, which prevented the deduction of any causality. Moreover, data on the nutritional intake of calcium were not obtained. However, a study conducted on healthy adults (age, 30–85 years) in Iceland with variable calcium intake (from <800 mg/day to >1200 mg/day) showed that subjects on high calcium intake (>1200 mg/day) did not have optimal serum PTH levels when vitamin D status was insufficient, whereas those with lower calcium intake (<800 mg/day) had ideal PTH levels if their vitamin D status was sufficient [28].

Another limitation was the use of corrected calcium levels rather than the ionized calcium levels, which is the form sensed by the calcium-sensing receptors on the parathyroid glands to increase or decrease PTH secretion accordingly. Magnesium deficiency among those with a blunted PTH response cannot be ruled out as the magnesium loading test, which may have better reflected body stores, was not performed.

The strength of this study stems from studying young women as PTH levels are known to increase with age [21]. All women studied were apparently healthy with normal renal function and not on any medications, which minimized confounding factors affecting PTH levels.

## 5. Conclusions

The results of this study suggest that a blunted PTH response to vitamin D deficiency is mainly observed in women with lower BMI, higher serum calcium levels, and higher 25(OH)D levels. This may indicate that these subjects are adapting to the lower 25(OH)D levels and maintaining normal calcium levels without the need to increase PTH secretion. The mechanisms underlying this adaptation are not clear, and future studies to explore these mechanisms are warranted. Future studies are also needed to confirm these findings and determine other factors that affect the relationship between vitamin D and PTH levels. It is debatable whether these women should be categorized as vitamin D deficient and receive supplementation, particularly if calcium homeostasis is maintained without increased PTH levels.

## Figures and Tables

**Table 1:** Distribution of age, anthropometric, and biochemical characteristics of the study group and those with or without a blunted PTH response.

Variables	All women (*n* = 315), median (IQR)	Blunted PTH response (*n* = 62), median (IQR)	Elevated PTH response (*n* = 253), median (IQR)	*P* value
Age (years)	31.0 (24.0–40.0)	26.5 (22.0–37.3)	32.0 (25.0–40.5)	0.041
Weight (kg)	67.0 (57.0–80.0)	61.0 (50.9–72.3)	68.0 (58.0–82.0)	<0.001
Height (cm)	157.0 (153.0–161.0)	156.5 (153.0–160.0)	157.0 (153.0–161.0)	0.597
BMI (kg/m^2^)	27.6 (22.4–32.1)	25.5 (20.0–29.3)	28.2 (23.1–33.2)	0.001
WC (cm)	79 (70–90)	73.5 (65.5–80.0)	80.0 (70.0–90.0)	<0.001
25(OH)D (nmol/L)	14.7 (10.8–21.0)	18.4 (12.6–25.3)	14.2 (10.0–19.9)	0.002
PTH (pmol/L)	10.0 (7.5–13.6)	6.0 (5.2–6.4)	10.9 (8.9–15.0)	<0.001
Albumin (g/L)	41.2 ± 3.6^*∗*^	41.6 ± 3.1	41.1 ± 3.7	0.308
C-Ca (mmol/L)	2.42 (2.29–2.66)	2.59 (2.35–2.75)	2.37 (2.29–2.63)	0.001
iPh (mmol/L)	1.25 (1.13–1.38)	1.30 (1.20–1.43)	1.24 (1.11–1.36)	0.005
Mg (mmol/L)	0.800 (0.700–0.800)	0.800 (0.700–0.800)	0.800 (0.700–0.800)	0.738
Creatinine (*μ*mol/L)	48.0 (42.8.55.0)	49.5 (41.0–57.5)	48.0 (43.0–54.8)	0.624

According to variable distribution, Mann–Whitney test or Student's *t*-test was used to compare the different variables among women with or without a blunted PTH response. ^*∗*^: mean ± standard deviation IQR, interquartile range; BMI, body mass index; WC, waist circumference; 25(OH)D, 25 hydroxyvitamin D; PTH, parathyroid hormone; C-Ca, corrected total serum calcium; iPh, serum inorganic phosphate; and Mg, serum magnesium.

**Table 2:** Spearman's correlation analysis between serum PTH level and other variables in all women with 25(OH)D <30 nmol/L.

	Age	BMI	WC	25(OH)D	C-Ca	iPh	Mg	Creatinine
PTH	0.165^*∗∗*^	0.279^*∗∗*^	0.252^*∗∗*^	−0.254^*∗∗*^	−0.218^*∗∗*^	−0.222^*∗∗*^	−0.007	0.068
Age	—	0.440^*∗∗*^	0.489^*∗∗*^	0.046	−0.059	−0.291^*∗∗*^	−0.013	0.110
BMI		—	0.836^*∗∗*^	−0.050	−0.052	−0.168^*∗∗*^	−0.112^*∗*^	0.096
WC			—	−0.067	−0.084	−0.172^*∗∗*^	−0.045	0.074
25(OH)D				—	0.016	0.031	−0.037	0.194^*∗∗*^
C-Ca					—	0.266^*∗∗*^	−0.218^*∗∗*^	0.000
iPh						—	0.051	−0.101
Mg							—	−0.052
Creatinine								—

Values in the table are Spearman's rho coefficients. ^*∗∗*^: correlation is significant at the 0.01 level; ^*∗*^: correlation is significant at the 0.05 level. BMI, body mass index; WC, waist circumference; 25(OH)D, 25 hydroxyvitamin D; PTH, parathyroid hormone; C-Ca, corrected total serum calcium; iPh, serum inorganic phosphate; and Mg, serum magnesium.

**Table 3:** Multivariate logistic regression of variables associated with a blunted PTH response.

Variable	Categories	OR	95% CI	*P* value
C-Ca	—	8.600	1.614–45.809	0.012
25(OH)D	—	1.068	1.014–1.124	0.013
BMI	—	0.925	0.867–0.987	0.018
iPh	—	3.991	0.583–27.303	0.158
Age	—	0.993	0.949–1.039	0.773
Mg	—	0.580	0.006–60.981	0.819
Smoking	No	Reference		
	Yes	0.884	0.161–4.844	0.887
Creatinine	—	1.001	0.974–1.029	0.935

OR, odds ratio; CI, confidence interval; C-Ca, corrected total serum calcium; 25(OH)D, 25 hydroxyvitamin D; BMI, body mass index; iPh, serum inorganic phosphate; and Mg, serum magnesium.

## Data Availability

The datasets used and/or analyzed during the current study are available from the corresponding author on reasonable request.
